# Topological Properties of Neuromorphic Nanowire Networks

**DOI:** 10.3389/fnins.2020.00184

**Published:** 2020-03-06

**Authors:** Alon Loeffler, Ruomin Zhu, Joel Hochstetter, Mike Li, Kaiwei Fu, Adrian Diaz-Alvarez, Tomonobu Nakayama, James M. Shine, Zdenka Kuncic

**Affiliations:** ^1^School of Physics, The University of Sydney, Sydney, NSW, Australia; ^2^Central Clinical School, The University of Sydney, Sydney, NSW, Australia; ^3^International Center for Materials Nanoarchitectonics (WPI-MANA), National Institute for Materials Science (NIMS), Tsukuba, Japan

**Keywords:** neuromorphic, atomic-switch networks, nanowires, topology, complex networks, structural connectivity, graph theory, artificial neural networks

## Abstract

Graph theory has been extensively applied to the topological mapping of complex networks, ranging from social networks to biological systems. Graph theory has increasingly been applied to neuroscience as a method to explore the fundamental structural and functional properties of human neural networks. Here, we apply graph theory to a model of a novel neuromorphic system constructed from self-assembled nanowires, whose structure and function may mimic that of human neural networks. Simulations of neuromorphic nanowire networks allow us to directly examine their topology at the individual nanowire–node scale. This type of investigation is currently extremely difficult experimentally. We then apply network cartographic approaches to compare neuromorphic nanowire networks with: random networks (including an untrained artificial neural network); grid-like networks and the structural network of *C. elegans*. Our results demonstrate that neuromorphic nanowire networks exhibit a small–world architecture similar to the biological system of *C. elegans*, and significantly different from random and grid-like networks. Furthermore, neuromorphic nanowire networks appear more segregated and modular than random, grid-like and simple biological networks and more clustered than artificial neural networks. Given the inextricable link between structure and function in neural networks, these results may have important implications for mimicking cognitive functions in neuromorphic nanowire networks.

## 1. Introduction

### 1.1. Graph Theory Applications

Graph theory is a framework used to represent complex networks mathematically, whereby network components are represented as nodes (*N*) and connections between components are represented as edges (*E*) (Boccaletti et al., 2006). Since the 1950s, graph theory has been applied to networks in a variety of fields, including social networks (Harary and Norman, 1953), progression of disease (Eubank et al., 2004; Mason and Verwoerd, 2007), transport networks (Wakabayashi and Iida, 1992), the internet (Albert et al., 2011), and many others. Graph theory has largely been employed to study the structure of networks, known as structural connectivity. Measures such as the path length (*PL*), clustering coefficient (*CCoeff*), participant coefficient (*PCoeff*), within-module degree z-Score (*MZ*), degree and small worldness (see Box 1 for definitions), are useful characterizations of the structural properties of a network (Strogatz, 2001; Estrada and Hatano, 2008; Grayson et al., 2016). In many cases, analyzing the structure of a network is the first step to understanding its function (Strogatz, 2001).

Box 1 **Graph Theory Terms**

**Clustering Coefficient (CCoeff)**: A measure of how much nodes in a graph tend to cluster together. This reflects the proportion of nodes connected to node *N* that are also connected to each other (Verweij et al., 2014).

**Degree (DEG)**: The number of edges connected to a node, *N*.

**Hubs**: Areas through which large amounts of information flow to reach from one part of a network to another (Types of hubs and non-hub nodes are described in **Figure 6**).

**Within-Module Degree z-Score (MZ)**: Measures how well connected a node is to other nodes in the same module (or cluster/community). This demonstrates whether the node is a hub in the network (i.e., much of the information flows through this node) (Guimerà and Amaral, 2005). Guimera and Nunes Amaral define MZ > 2.5 as hub-like nodes, and MZ < 2.5 as non-hub nodes.

**Modularity**: A measure of network segregation into distinct modules (or clusters/communities) that have sparse connections between each module (Cohen and D'Esposito, 2016).

**Participant Coefficient (PCoeff)**: Measures how homogeneous a node's edges are distributed across modules (or clusters/communities). Nodes are divided into two classes: (1) High PCoeff: connector nodes with many global edges across modules (strong between-module and weak within-module connectivity Rubinov and Sporns, 2010; Cohen and D'Esposito, 2016); and (2) Low PCoeff: provincial/local nodes with mostly edges that connect nodes within a module (strong within-module and weak between-module connectivity) (Joyce et al., 2010; Van Diessen et al., 2014; Bertolero et al., 2015).

**Path Length (PL)**: Measures the minimal number of edges of all possible node connections in a network (Van Diessen et al., 2014; Verweij et al., 2014).

**Small–worldness**: A type of network architecture in which local clustering is combined with short path length. This architecture offers important advantages for network functionality, ranging from synchronizability to information flow (Oliveira et al., 2014; Muldoon et al., 2016).

**Small–world Propensity**: Introduced by (Muldoon et al., 2016), used to account for potential variations in connection strength in a network, by measuring how clustering and path length differ from random and grid-like networks.

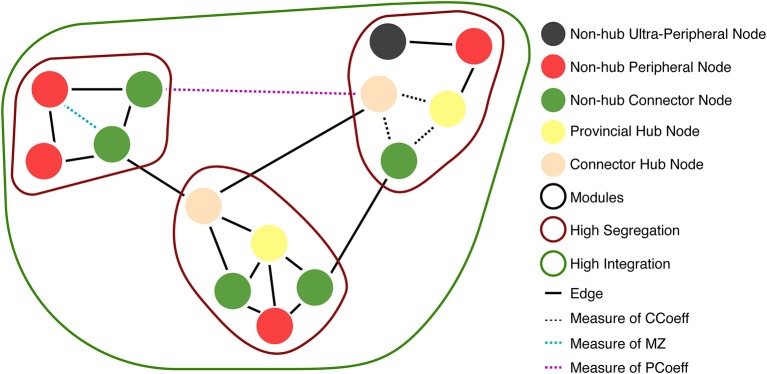

Graph theory measures have been applied to the study of biological networks, including the brain structure of organisms such as the neural networks of *C. elegans* (Achacoso and Yamamoto, 1991; Yan et al., 2017) and Macaque monkeys (Achard et al., 2006), in attempt to better understand their function. Biological networks typically demonstrate a small–world architecture (see Box 1 for definition). Small–worldness has been shown to allow for high efficiency of synchronized and parallel information transfer between neural regions (Bullmore and Sporns, 2009). Within such a system, shorter paths from node to node (with few longer sparse connections) may provide more efficient communication across an entire system, thereby facilitating dynamical processes that require global coordination and information flow (Watts and Strogatz, 1998; Strogatz, 2001). For instance, regions with short path length and high clustering coefficient confer an ability to transfer information quickly between a large number of nodes. Contrastingly, areas with long path lengths and low clustering may allow for sparse connections between individual clusters in a network, resulting in a slower spread of information over greater distance (Strogatz, 2001; Bullmore and Sporns, 2012; Muldoon et al., 2016). Understanding these distinct structural features within biological neural networks has allowed researchers to infer that such networks may utilize different structural properties to communicate under separate time scales (e.g., fast local synchronization within dense regions and slow global communication between dense regions; Chow and Kokotovic, 1985; Tahmassebi et al., 2017).

The commencement of the Human Connectome Project in 2005 (Sporns et al., 2005) has driven a surge in techniques and studies to map the structure and function of the human brain network (Sporns et al., 2002; Bertolero et al., 2015; Farahani et al., 2019; Gilson et al., 2019). Many such studies apply graph theory to analyse the connectivity within and between regions of the brain (Bullmore and Sporns, 2009). While the networks of simple organisms such as *C. elegans* are composed of only 270–300 individual neurons (Yan et al., 2017), the human brain is a much larger network, composed of tens of billions of neurons (although the exact number is contested), each of which has around ten thousand synapses (Koch, 2004; Shepherd, 2004). The sheer number of neural components makes it extremely difficult to model human neural networks graphically. Therefore, much of the graph theory analysis on human neural networks is applied to large collection of neurons, or even entire regions of the brain (e.g., Bassett and Bullmore, 2006; Gilson et al., 2019).

### 1.2. Neuromorphic Systems: Mimicking the Brain in Hardware

In parallel to developments in neuroscience, the engineering community has spurred the development of neuromorphic systems that can mimic the function of human neurons in hardware (Vianello et al., 2017). Carver Mead's pioneering efforts to emulate biological information processing using analog circuits (instead of logic gates used in digital computing) and leveraging the inherent device physics of Metal Oxide Semiconductor Field Effect Transistors (MOSFETs) established a new paradigm in computing hardware (Mead, 1990). Today, neuromorphic computing encompasses the use of novel nanotechnologies such as non-volatile memory devices and memristors (memory-resistors) that can mimic synapse-like memory and spiking temporal characteristics (Yang et al., 2013; Burr et al., 2017; Ziegler et al., 2018; Roy et al., 2019). Because of their unconventional “beyond von Neumann” architecture, which substantially reduces power requirements, such devices are also attractive for implementing Artificial Neural Network (ANN) algorithms, which require computationally-intensive training to learn input-output relationships, thereby mimicking neurons and synaptic connections in software (Xu et al., 2018).

Similarly, neuromorphic chips [e.g., IBM's TrueNorth (Merolla et al., 2014; Akopyan et al., 2015) and Intel's Loihi (Davies et al., 2018)] have been developed specifically as ANN accelerators, although their neuromorphic hardware attributes are limited to the integration of processing and memory to reduce power requirements. More generally, a limitation of neuromorphic in-memory computing hardware systems is their restriction to a regularized grid-like array structure that emphasizes the role of individual synapse-like elements (e.g., memristors), rather than the network architecture as a whole.

This limits potential advantages arising from structure–function integration in a distributed network, such as in a small–world architecture seen in biological neural networks (Bullmore and Sporns, 2009; Chialvo, 2010). It is likely that due to their conventional grid-like array structure, most neuromorphic systems lack the emergent dynamical properties that are characteristic of neural network circuitry (e.g., memory, learning, and even intelligence). Such emergent properties are attributed to the complexity of neural networks and the interplay between structure and function (Hagmann et al., 2008; Chialvo, 2010; Bassett and Gazzaniga, 2011). It is important to note that factors other than topology may influence emergent behavior (e.g., learning rules specifically designed for ability acquisition; Chollet, 2019). However, much of the literature exploring emergence in complex systems, including biological networks, emphasizes the role of topology, and structural properties as key to understanding emergence (Angeline, 1994; Chialvo, 2010; Pascual-García, 2016; Dumitrescu et al., 2017).

We previously introduced a novel neuromorphic system comprised of self–assembled nanowires whose structure and function (in response to electrical stimulation) mimic that of biological neural networks (Kuncic et al., 2018; Diaz-Alvarez et al., 2019). In these networks, each junction between nanowires provides a non-linear synaptic function in a similar manner as an atomic switch (Terabe et al., 2005; Ohno et al., 2011). Rather than focusing on the controllability of individual synapses like ANNs or other neuromorphic systems, our Atomic Switch-like Networks (ASNs) mimic the complex topology of biological neural networks, by mimicking biological self–assembly to form similarly complex networks comprised of nanowires (synthetic neurons) and junctions (synthetic synapses) (Stieg et al., 2012; Diaz-Alvarez et al., 2019).

Previous studies have shown that ASNs exhibit emergent properties such as non-linear dynamics, recurrence and capacity for learning, which arise from the complexity of the networks, as well as the properties of the atomic switch-like junctions (Terabe et al., 2005; Avizienis et al., 2012; Kuncic et al., 2018). Such properties are essential for brain-like function (Avizienis et al., 2012). However, due to the complexity of ASNs, it is highly difficult to understand or predict the impact and interactions of the networks' structure and functions from experimental data alone. Furthermore, due to the networks' self-assembled structure, it is experimentally difficult to control the topology to measure how it influences dynamics. It is also extremely difficult to use imaging-based techniques such as or electron microscopy (e.g., White et al., 1986; Eberle and Zeidler, 2018) reconstructions to unpack the structural connectivity of ASNs, as it is impossible to tell whether or not intersecting wires form a junction between them. We therefore have developed a computational model that simulates the structure experimental ASNs, based on functional, experimental validation (Kuncic et al., 2018; Diaz-Alvarez et al., 2019). For the purposes of the present study, we use this model solely to construct simulated self-assembled networks for structural analysis. ASNs are made of a fixed nanowire structure that does not change under electrical activation. Our simulations allow us to visualize each wire and connection individually in a graphical representation, and to easily alter them, either by changing the positioning and lengths of individual wires and junctions, or manipulating the density and dispersion of the networks. Consequently, our model enables us to examine the structural properties of specific sections of the network, which is currently impossible to do experimentally, as well as different realizations of nanowire networks.

Here, we apply graph theory measures to simulated ASNs with varying topologies. This allows us to examine the topological properties of ASNs, and compare them with a range of real-world networks. These include the simple organism *C. elegans*, as well as random and grid-like networks.

## 2. Methods

### 2.1. Construction of Simulated ASNs

**Figure 1:**
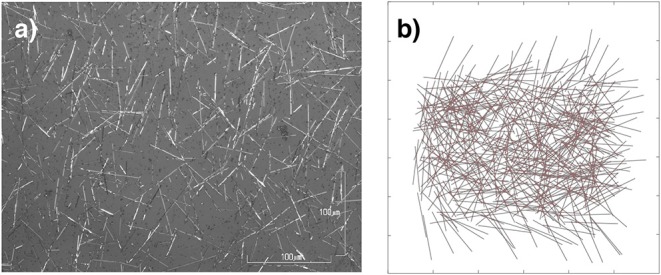
Neuromorphic nanowire networks. **(a)** Optical microscopy image of an actual self–assembled network of nanowires. The length of wires varies from~6 to 50μm in this image. **(b)** Simulated 500 nw (6,065 junction) network generated by our model. The length of wires in the simulated networks varies from 6 to 9μm.

To explore the topology of ASNs, we generated multiple networks with different structural parameters (see Figure 1 for visualizations). Hardware ASNs acquire a complex network structure through bottom-up self-assembly (Avizienis et al., 2012; Stieg et al., 2012; Diaz-Alvarez et al., 2019), similar to neural network growth in the brain. To simulate this self-assembly, we modeled nanowires as 1D objects of length uniformly drawn from a normal distribution of specified average wire length (mean of distribution, ranging from 6 to 9 μm) and wire dispersion (ratio of standard deviation to the mean, ranging from 0 to 50%). These wires were randomly placed within a 2D plane of fixed size (30 × 30 μm), with horizontal and vertical positions of the wire centers generated from a uniform spatial distribution. The angular orientation of each wire was generated from a uniform distribution on 2π. A junction was modeled at each intersection point between nanowires (Kuncic et al., 2018; Diaz-Alvarez et al., 2019). The connectivity was mapped to a graph adjacency matrix representation with nodes corresponding to nanowires and edges corresponding to junctions. In real networks, not every intersection between wires need necessarily form a junction. It is, however, practically difficult to determine where individual junctions exist in the self-assembled networks (Diaz-Alvarez et al., 2019). In our simulated networks, the simplifying assumption that all intersections result in junctions has negligible effect on network functionality when compared to experimental measurements of hardware ASNs (see Supplementary Materials).

For each of the networks, the following parameters were varied: number of nanowires (i.e., 100, 500, 1, 000, or 2, 000 nws), average nanowire length (6 – 9 μm), and dispersion of wire length (0, 10, 20, and 50% of average nanowire length). Using this process, we generated a total of 39 different combinations of networks. All simulated networks were constructed in Matlab v2018a and Python v3.7.3. All structural connectivity measures were taken from the open-source Brain Connectivity Toolbox (Rubinov and Sporns, 2010) and NetworkX (Hagberg et al., 2008) packages.

**Figure 2:**
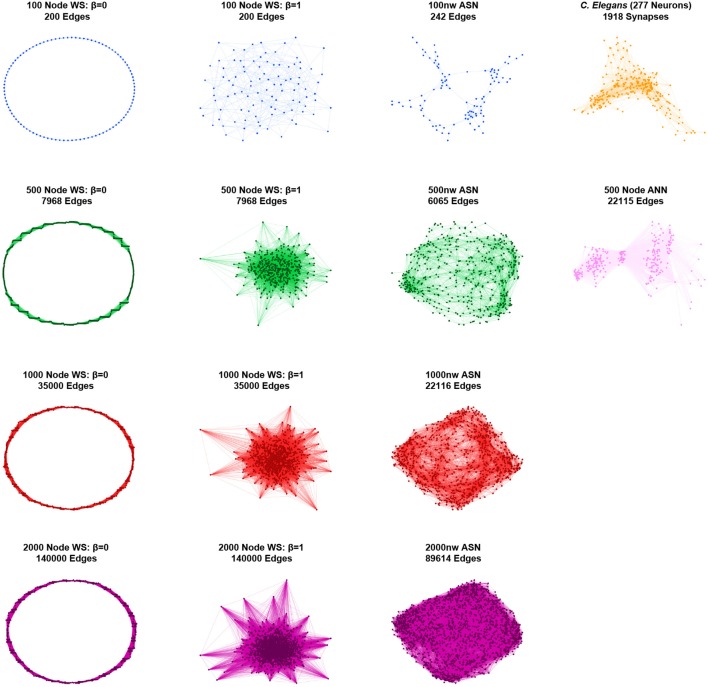
Graphical representations of sample networks: Grid-like Watts-Strogatz (WS) networks (β = 0; left); random Watts-Strogatz networks (β = 1; center-left); ASNs (center-right); *C. elegans* and fully-connected ANN networks (right). Nodes represent nanowires (or neurons for *C. elegans* and ANN), while edges represent junctions (or synapses for *C. elegans* and virtual synapses for ANN).

To contextualize the structural connectivity of our ASNs, we simulated the topology of Watts-Strogatz networks ranging from random to grid-like, and *C. elegans*. Graph theory measures were applied to the connectivity data of each ASN, as well as to each of the Watts-Strogatz and *C. elegans* networks (see Figure 2 for graphical representations of all networks). We also included a fully-connected ANN similar to a random Watts-Strogatz network. Next, we compared global clustering coefficient and average path lengths (Watts and Strogatz, 1998). We also calculated the small–world propensity values for each network to establish an unbiased (see Box 1) measure of small–worldness in all networks (Muldoon et al., 2016). Finally, we mapped 100 and 500 nw ASNs, as well as *C. elegans* and correspondingly sized random and grid-like WS networks, on the Guimerà and Amaral (2005) cartographic plane to compare participant coefficient and within-module degree z-score. This allowed us to examine the modularity and integration of the networks (Guimerà and Amaral, 2005; Power et al., 2013; Bertolero et al., 2015).

### 2.2. Construction of Random and Grid-Like Watts-Strogatz Networks

To create a series of Watts-Strogatz networks, we first created a ring lattice with *N* nodes of mean degree *2k*, where *2k* = mean degree of the corresponding ASN with *N* nodes. In the Watts-Strogatz networks, each node is connected to its *k* nearest neighbors on either side. For each edge, *E*, in the graph, we then rewire the target node to *k* other nodes in the network with probability β. When β = 0, no edges are rewired and the model returns a locally-clustered ring lattice. We term this network grid-like, as its non-graphical representation is formed from a grid-shaped lattice. In contrast, when β = 1, all of the edges are rewired and the ring lattice is transformed into a random graph (MathWorks, 2016). We varied β from 0 to 1 in steps of 0.05, leaving 21 networks ranging from completely Grid-Like (β = 0) to completely Random (β = 1), for each size *N*. A β of 0.2 is denoted as displaying “small–world” characteristics (Watts and Strogatz, 1998). Furthermore, to compare ASNs with a WS random-like ANN model, we constructed a 5-layer ANN, with 10 input nodes, 10 output nodes and 160 nodes in each middle layer. Every node in each layer is connected to every node in its parent and child layers (hence the term “fully-connected”). However, no nodes are connected within layers.

### 2.3. Construction of *C. elegans* Networks

Neuronal connectivity data of the simple nematode *C. elegans* (277 neurons and 2,105 synaptic connections) was adapted from Achacoso and Yamamoto (1991), and electron microscope reconstructions by White et al. (1986).

## 3. Results

### 3.1. Small–Worldness

We compared the structures of multiple unique ASNs across four sizes (a total of 39 networks comprised of 100, 500, 1,000, or 2,000 nanowires) with a fully-connected ANN, a *C. elegans* network, and Watts-Strogatz random/grid-like networks across four sizes and 21 varying β parameters (one network for each β, and for each size). See Table 1 for a full statistical description of each network.

**Table 1:** Mean and Standard Deviation (SD) for ASNs, WS random networks, WS grid-like networks of different sizes, and *C. elegans*.

	**ASNs**				**WS β = 1 (Random)**				**WS β = 0.5**				**WS β = 0 (Grid-like)**				***C. elegans***
Number of Nodes	100	500	1,000	2,000	100	500	1,000	2,000	100	500	1,000	2,000	100	500	1,000	2,000	277
Mean (PL)	3.65	2.96	2.77	2.65	2.74	2.05	1.94	1.93	2.8	2.11	1.95	1.93	8.76	8.27	7.64	7.65	2.69
SD (PL)	0.13	0.07	0.08	0.06	0.76	0.42	0.27	0.26	0.79	0.47	0.29	0.26	4.77	4.49	4.12	4.12	0.79
Mean (CCoeff)	0.39	0.39	0.4	0.4	0.05	0.06	0.07	0.07	0.1	0.13	0.14	0.14	0.6	0.73	0.74	0.74	0.2
SD (CCoeff)	0.01	0.01	0	0													
Mean (PCoeff)	0.22	0.29	0.31	0.31	0.57	0.85	0.87	0.89	0.52	0.7	0.7	0.69	0.2	0.16	0.17	0.19	0.41
SD (PCoeff)	0.23	0.22	0.22	0.23	0.17	0.03	0.02	0.01	0.18	0.09	0.07	0.06	0.21	0.2	0.2	0.2	0.25
Mean (MZ)	0	0	0	0	0	0	0	0	0	0	0	0	0	0	0	0	0
SD (MZ)	0.97	0.99	1	1	0.97	0.99	1	1	0.97	0.99	1	1	0.96	0.99	1	1	0.99
Mean (Small–world Prop)	0.69	0.68	0.67	0.67	0.29	0.29	0.29	0.29	0.29	0.29	0.29	0.29	0.37	0.37	0.37	0.37	0.55
SD (Small–world Prop)	0.04	0.02	0.01	0.01													

**Figure 3:**
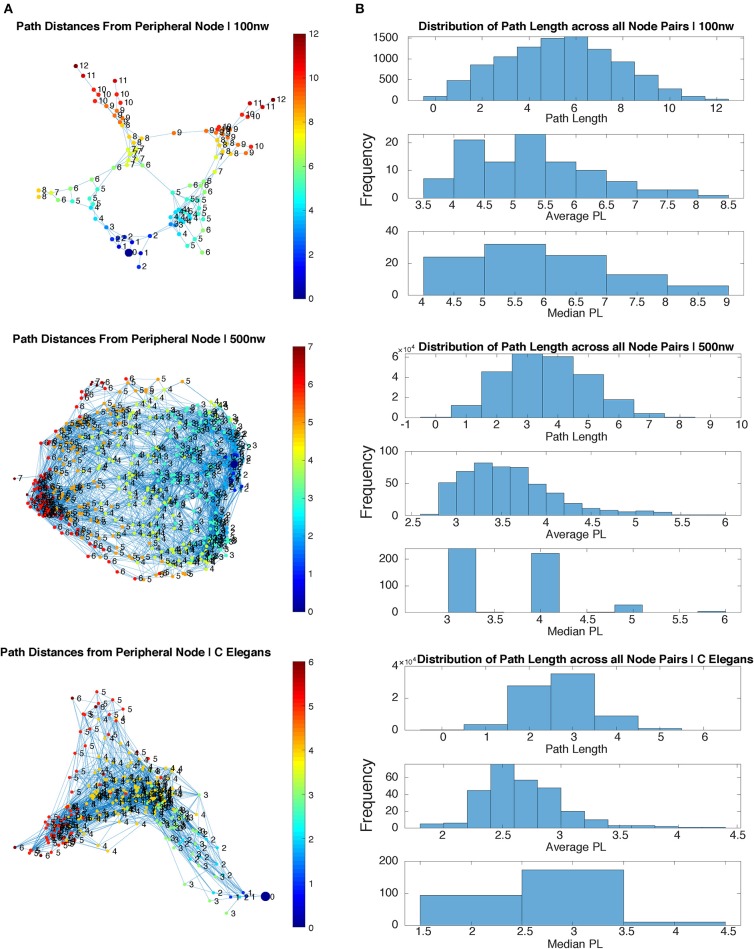
Path length comparison of sample 100 and 500 nw ASNs with *C. elegans*. **(A)** Path length of each node from a randomly selected peripheral node for sample 100, 500 nw and *C. elegans* networks. **(B)** Distribution of path lengths from all node pairs in each sample network, including the average and median path length distributions, for 100, 500 nw and *C. elegans* networks.

**Figure 4:**
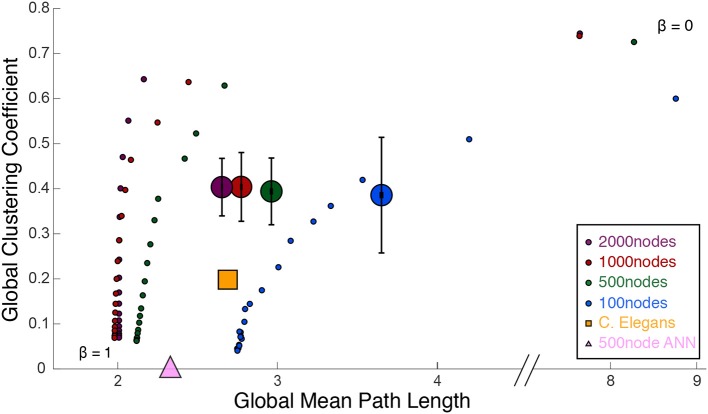
Watts and Strogatz (1998) cartographic plane: global clustering coefficient and global mean path length. Each large dot represents 39 ASNs of varying parameters, with colors representing the network size (number of nanowire nodes). The small dots are Watts-Strogatz networks, rewired from completely random (β = 1) to grid-like (β = 0). Beta values decrease from bottom to top. The large yellow square measures the *C. elegans* network, and the large pink triangle is a 500-node fully-connected ANN.

Figure 3 shows a comparison of path lengths and path distances between 100 and 500 nw ASNs, and a *C. elegans* network. Figure 4 shows a comparison of global mean path length and global clustering coefficient for each of the networks studied. Larger ASNs have similar mean path length to *C. elegans*, but higher clustering. However, ASN networks of similar size to *C. elegans* have a higher average path length.

ASNs are also more clustered and have a longer mean path length than random WS networks (β = 1). Compared to grid-like WS networks (β = 0), ASNs tend to be less clustered with generally shorter path lengths. Compared to a fully–connected ANN of 500 nodes, ASNs display much higher clustering, and longer path lengths.

Using path length and clustering coefficient to estimate small–worldness Watts and Strogatz (1998), ASNs would fall in the small–world category, with relatively low path length and high clustering. Recently a measure called small–world propensity has been employed to consider potential drawbacks of the Watts–Strogatz method (see Box 1; Muldoon et al., 2016).

**Figure 5:**
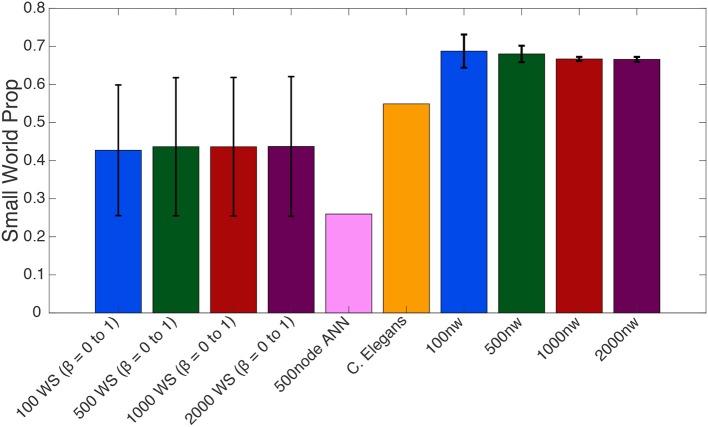
Average small–world propensity values for Watts-Strogatz (WS), 500-node ANN, *C. elegans* and ASNs of varying sizes (number of nanowires, nw). Averages for WS were taken across all 21 β parameters from 0 to 1, with error bars reflecting standard deviation across β parameters. Averages for ASNs were taken from 39 networks with varying parameters as described in the methods section, with error bars reflecting standard deviation across network parameters.

One-way ANOVAs were conducted to compare the small–world propensity of 100 nw ASNs with 100 node WS random-like, 100 node WS grid-like and *C. elegans* networks. There was a significant difference between groups [*F*_(3,10)_ = 47.16, *p* < 0.001] (where *F* is the ratio of mean square values of each group). *Post-hoc* analysis using the Bonferonni correction for multiple comparisons indicated that 100 nw ASNs had higher small–world propensity [*Mean*(*M*) = 0.69, *Standard* *Deviation*(*SD*) = 0.04] than random networks (*M* = 0.29, *SD* = 0) and grid-like networks (*M* = 0.29, *SD* = 0), but there was no significant difference between ASNs and *C. elegans* (*M* = 0.55, *SD* = 0; see Supplementary Materials for boxplots and multiple comparison graphs). We repeated these ANOVAs for 500 nw ASNs and 500 node WS networks [*F*_(3,9)_ = 182.16, *p* < 0.001]. *Post-hoc* analysis indicated that 500 nw ASNs (*M* = 0.68, *SD* = 0.02) had higher small–world propensity than random networks (*M* = 0.29, *SD* = 0), grid-like networks (*M* = 0.29, *SD* = 0) and *C. elegans* (*M* = 0.55, *SD* = 0). Figure 5 shows a visual difference between ASNs and other networks.

### 3.2. Modularity and Integration

**Figure 6:**
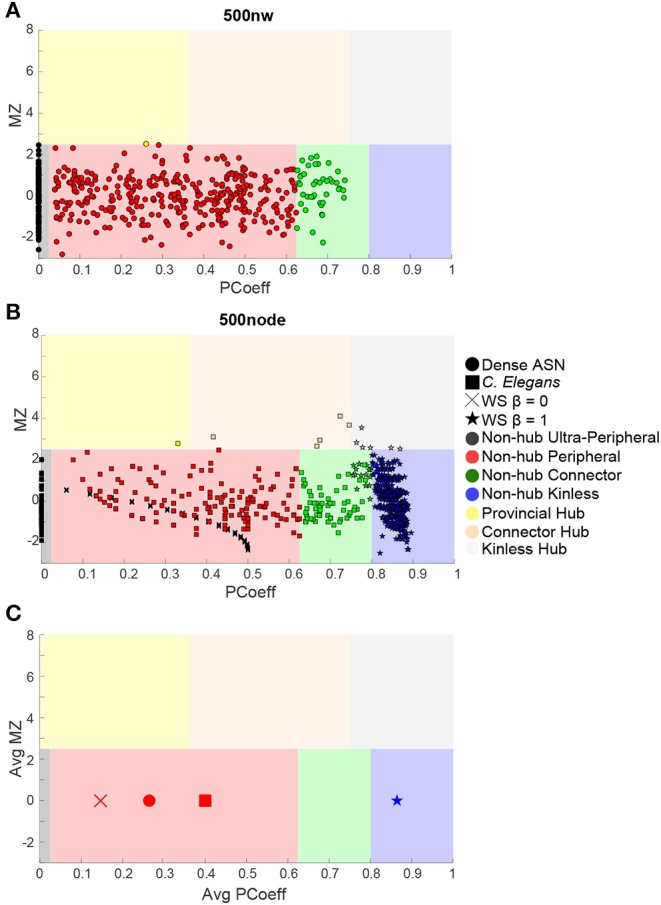
Guimera-Amaral (2005) Cartographic-Space: Within-Module Degree z-Score (MZ) and Participant Coefficient (PCoeff). The dark-gray region (bottom left) refers to ultra-peripheral nodes (i.e., nodes with only one or few connections within a module); The light red region refers to peripheral nodes (i.e., nodes that are non-hubs and are only connected within a module); The green region refers to non-hub connector nodes (i.e., nodes that are connected to other modules but are not hubs); The blue region (bottom right) refers to non-hub kinless nodes (i.e., non-hub nodes not belonging to a particular module); The yellow region (top left) refers to provincial hubs (i.e., hub nodes that are highly connected within a module, but not between modules); The cream region refers to connector hubs (i.e., nodes that are highly connected between modules, but not necessarily within modules); the light-gray region (top right) refers to kinless hubs (i.e., hubs not belonging to a particular module). Regions are adapted from Guimerà and Amaral (2005). See Box 1 for example nodes. **(A)** 500 nw ASN networks. Each colored dot is the mean MZ and PCoeff for the most dense (Avg DEG = 40.66) variations of ASNs. **(B)** 500 node WS networks and *C. elegans* networks. Squares represent the MZ and PCoeff for *C. elegans*. Stars and crosses represent the MZ and PCoeff values for WS networks with β = 0 and 1, respectively. **(C)** Average PCoeff and MZ scores for each type of network.

We used MZ and PCoeff measures to plot ASNs on a Guimerà and Amaral (2005) cartographic space (see Figure 6A for 100 and 500 nw ASN values, and Supplementary Figure 5 for 1,000 and 2,000 nw ASN values). Briefly, this involves calculating the modular assignment of each node (see Box 1), and then estimating each nodes' topological role, relative to the modular assignment: high MZ = high within-node connection (segregation) and high PCoeff = high between-node connection (integration). When combined, these measures exhibit the modularity and hub characteristics of a network. Each region in this space classifies a node in a network as a specific type. Almost all the nodes in all sizes of ASNs were categorized as ultra-peripheral (PCoeff = 0), peripheral (MZ < 2.5, 0 < PCoeff < 0.62), and non-hub connector regions (MZ < 2.5, 0.62 < PCoeff < 0.80). There were also a very few nodes that fell in the provincial hub region (MZ > 2.5, and 0 < PCoeff < 0.30).

We compared the PCoeff and MZ of ASNs to both WS networks, and a biological system such as the *C. elegans* (see Figure 6B). One-way ANOVAs were conducted to compare the PCoeff and MZ of 100 nw ASNs with 100 node WS random-like, 100 node WS grid-like and *C. elegans* networks. There was a significant difference between groups [*F*_(3,1,517)_ = 112.64, *p* < 0.001] for PCoeff, but there was no significant difference for MZ. *Post-hoc* analysis using the Bonferonni correction for multiple comparisons indicated that 100 nw ASNs had lower PCoeff (*M* = 0.22, *SD* = 0.23) than *C. elegans* (*M* = 0.41, *SD* = 0.21) and random networks (*M* = 0.57, *SD* = 0.17), but there was no significant difference between ASNs and grid-like networks. We repeated these ANOVAs for 500 nw ASNs and 500 node WS networks, and the results were largely unchanged (see Supplementary Materials).

The structures of WS random-type networks tend to have higher PCoeff values (see Table 1 for means and standard deviations), mainly in the PCoeff > 0.8 region (non-hub kinless nodes). They also have some examples of MZ > 2.5 in the connector and kinless hub regions, but mainly MZ < 2.5. WS grid-like networks have lower PCoeff values, typically limited to ultra-peripheral and peripheral regions. *C. elegans* networks cover a greater portion of the cartographic space, although most of the nodes tend to fall within the peripheral and non-hub regions (see Guimerà and Amaral, 2005 for more examples of biological PCoeff/MZ distributions).

## 4. Discussion

ASNs exhibit a small–world structure, characterized by relatively short mean path length, alongside high clustering (Sporns et al., 2002; Sporns and Zwi, 2004). When compared with random or grid-like Watts–Strogatz networks, ASNs demonstrate more biological-like small–worldness features. While both random networks and ASNs have short path lengths, ASNs show higher clustering. In studies on human neural networks, it has been suggested that a small–world network is ideal, for example, for synchronizing neural activity between brain regions (Latora and Marchiori, 2001; Reijneveld et al., 2007; Verweij et al., 2014). In turn, this reflects the capacity for high global efficiency of parallel information transfer between such regions (Bullmore and Sporns, 2009). ASNs may therefore have capacity for efficient, synchronized and parallel information transfer across the entire network, similar to that of biological systems.

However, the structure of wiring within and between regions/clusters, as highlighted by PCoeff and MZ measures, may be different from biological systems such as *C. elegans* (see Supplementary Figure 3 for comparison with human node types). In biological neural networks, PCoeff and MZ are used to identify whether particular nodes play a hub-like role in the network. Hubs are central areas through which large amounts of information is trafficked to reach different parts of a network (van den Heuvel and Sporns, 2013). They are characterized by high connectivity to other network regions, as well as central positioning in the network. MZ scores have been used to denote hub status (e.g., z-Score < 2.5), while PCoeff values are used to classify the type of hub (Guimerà and Amaral, 2005; Joyce et al., 2010). Our results are consistent with previous studies showing that nodes in *C. elegans* have many peripheral and non-hub connector nodes, but also some hub-type provincial and connector nodes (Achacoso and Yamamoto, 1991; Guimerà and Amaral, 2005; Power et al., 2013). Such networks maintain a balance between integration and segregation of modules. In contrast, random WS networks are largely comprised of highly integrated, non-hub nodes, with a few hub-type nodes. This reflects a network with few modules. Grid-like WS networks are entirely comprised of non-hub, ultra-peripheral/peripheral type nodes, with very little integration or even modularity, as they have no connector nodes to connect between any modules that may exist.

How do ASNs fit within this space? Our results suggest that ASNs have a high proportion of peripheral, non-hub type nodes, similar to grid-like graphs. However, ASNs also have many non-hub connector nodes, which grid-like graphs lack. This means that ASNs are highly segregated, but also have many connections between modules, although they are weaker than within-module connections. Therefore they also have higher modularity than *C. elegans*. Random networks, on the other hand are highly integrated and have very few connector or peripheral nodes. Therefore ASNs have greater segregation than random networks, and higher modularity than both random and grid-like networks. ASNs involve a balance between integration and segregation, that is biased toward the presence of highly clustered, tight-knit modules with sparse inter-connectivity.

However the modularity and segregation of ASNs do not seem to reflect that of an organism like *C. elegans*. The nematode network has a greater balance between segregation and integration than ASNs, although likely with less modularity. Even ASNs that are highly dense only have a few hub-type nodes, meaning that most of the network's capacity to transfer information occurs in segregated modules, with sparse links between modules. Networks like the *C. elegans* would likely have fewer modules, with more central hub-type nodes that are responsible for directing information flow to and from the segregated modules of the network (hence the term hub).

Optimization of the structure of ASNs to represent biological-like networks may be desirable in the future, to allow for more biological-like capacities. For instance, increasing the size of the networks, and allowing for a greater balance between sparse and dense connections may allow for a more equivalent distribution of MZ and PCoeff scores, as well as increasing small–worldness even more. If these parameters are changed, it may be possible to construct nanowire networks that are even more representative of a biological system. However, it may be that ASNs currently demonstrate similar functionality to biological organisms, but with a uniquely highly modular and segregated structure that has more emphasis on peripheral-type nodes. In such a case, nodes within a particular module or cluster may communicate more within that module than with nodes outside it, yet still produce dynamics that are similar to biological systems (Kuncic et al., 2018). Due to the high small–worldness that ASNs demonstrate, it may be possible that these types of networks place much less importance on hub-type nodes or regions, as many other small–world complex networks do (e.g., Guimerà and Amaral, 2005; van den Heuvel and Sporns, 2013; Verweij et al., 2014). We plan to investigate the functional connectivity of ASNs in a future study, to understand how similar the interplay between structure and function in these networks may be to biological systems and other real-world networks.

### 4.1. Conclusion

Neuromorphic nanowire networks demonstrate a small–world architecture that is similar to the biological system of *C. elegans*, and is distinct from random or grid-like networks (including untrained artificial neural networks). However, they also appear to be comprised of nodes that are equivalent to peripheral or non-hub nodes in a biological system, while being more segregated and modular, and less reliant on hubs of information flow. In future studies, investigating the functional properties of neuromorphic nanowire networks under electrical activation, coupled with altering the topology of these networks, will provide new insights into the interplay between structural and functional connectivity in a way that is extremely difficult experimentally. This may bring us closer to better understanding the physical components that may give rise to emergent dynamical behaviors of neural-network-like structures; behaviors that, in turn, enable cognitive functions such as learning and memory, or even intelligence.

## Data Availability Statement

The code used to generate the datasets analyzed for this study can be found on Github. Datasets are also available on request from the corresponding author.

## Author Contributions

Data collection, writing, and editing was conducted mainly by AL, with support from RZ, JH, ML, and KF. AD-A and TN helped with code and experimental verification of the simulation model. JS and ZK supervised the project and provided writing, editing, and feedback for the manuscript.

### Conflict of Interest

The authors declare that the research was conducted in the absence of any commercial or financial relationships that could be construed as a potential conflict of interest.
