# Supplementary figures and images for: Topological Properties of Neuromorphic Nanowire Networks
Source: Front Neurosci. 2020 Mar 6;14:184. doi: 10.3389/fnins.2020.00184 (PMC7069063; doi:10.3389/fnins.2020.00184)

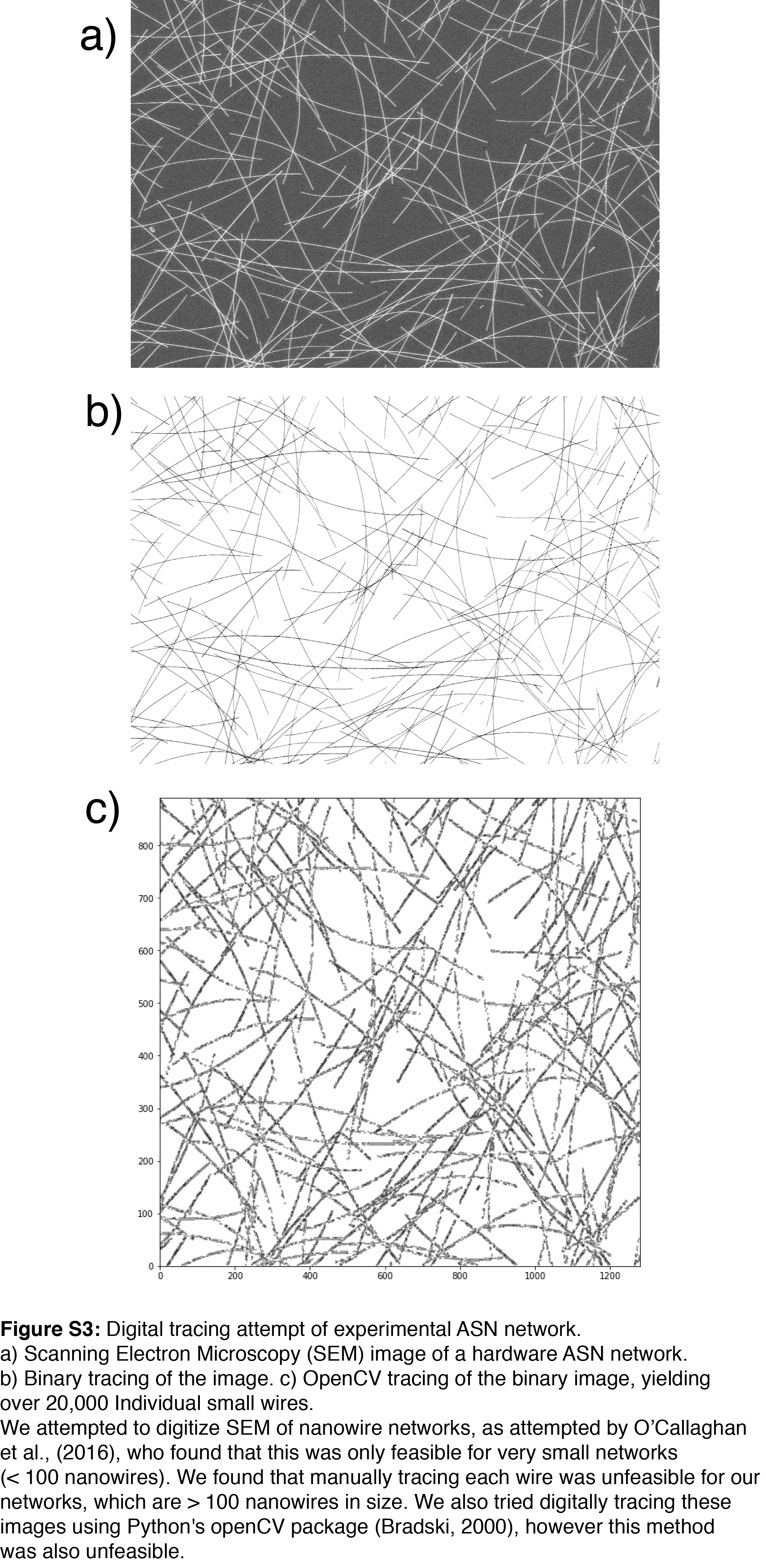

Supplement: Supplementary file 3 [file Image_3.JPG]
